# An Insight on Flavonoids and Flavonoids incorporated Nano-formulations in the Management of Diabetes Mellitus

**DOI:** 10.2174/0115733998354264250402054755

**Published:** 2025-04-16

**Authors:** Madhavi Patel, Priya Shah, Sudarshan Singh, Bhupendra G Prajapati

**Affiliations:** 1 Parul Institute of Pharmacy, Parul University, Limda, Vadodara, Gujarat, 391760, India;; 2 L.J. Institute of Pharmacy, L.J. University, Sarkhej, Ahmedabad, Gujarat, 382210, India;; 3 Faculty of Pharmacy, Chiang Mai University, Chiang Mai, 50200, Thailand;; 4 Office of Research Administration, Chiang Mai University, Chiang Mai, 50200, Thailand;; 5 Shree S. K. Patel College of Pharmaceutical Education and Research, Ganpat University, Kherva, 384012, India;; 6 Faculty of Pharmacy, Silpakorn University, Nakhon Pathom, 73000, Thailand

**Keywords:** Bioactive compounds, flavonoids, natural product, nano-formulations, diabetes mellitus, solid lipid nanoparticles

## Abstract

Diabetes mellitus is a worldwide health challenge and imposes a considerable strain on healthcare systems. By 2035, it is anticipated that around 592 million adults will be impacted by diabetes as a result of causes like aging, rapid population expansion, urbanization, obesity, lifestyle modifications, and heightened intake of calorie-dense, fatty, and fast meals. Despite the availability of several pharmaceutical therapies, they often come with higher expenses and adverse side effects, making them inaccessible to a large portion of the population. Bioactive compounds, especially flavonoids, are emerging as potential alternatives for diabetes management. Flavonoids, a category of polyphenolic chemicals included in fruits, vegetables, and various plant-derived foods, provide numerous therapeutic advantages. In addition to their glucose-lowering benefits, they increase antioxidant defence, improve insulin efficacy, and diminish the risk of cardiovascular problems. The clinical application of flavonoids is hindered by issues like poor solubility, low bioavailability, and complicated metabolism. This review explores the integration of nanotechnology-based delivery technologies to address these limitations. Advanced nano-formulations, such as polymeric nanoparticles, liposomes, micelles, and solid lipid nanoparticles, improve the solubility, stability, and absorption of flavonoids while facilitating targeted delivery and prolonged therapeutic benefits. The review highlights the efficacy of flavonoid-based nano-formulations in efficiently managing diabetes mellitus and its related problems. Moreover, it highlights the need for ongoing research to enhance these formulations and explore the molecular pathways that underlie their antidiabetic efficacy. This approach demonstrates the significance of nanotechnology in transforming diabetes care and enhancing patient outcomes.

## 1 INTRODUCTION

Diabetes mellitus is a universal disorder spreading at an alarming rate identified by rising in blood glucose level because of unusual metabolism of fats, proteins and carbohydrates in the peripheral tissues, for example adipose, liver and skeletal muscle tissue. Insufficient insulin secretion by pancreatic β-cells in the islets of Langerhans further exacerbates the condition [1]. The increase in the blood sugar level triggers a cascade of metabolic disorders such as retinopathy, nephropathy, neuropathy, cardiac arrest, and hypertension because of damage to various organs. Because of factors such as an aging population, rapid population growth, increasing urbanization, a high prevalence of obesity, and the proliferation of calorie-rich, fatty, and fast food, the International Diabetes Federation predicts that around 592 million persons will get diabetes by 2035 [2, 3]. Diabetes is broadly categorized into three types:(i) Type I diabetes/ Insulin-dependent diabetes [4], which is caused by the autoimmune destruction of pancreatic β-cells. It typically manifests in childhood or adolescence and affects approximately 10% of diabetes cases [5]. (ii) Type II diabetes/non-insulin-dependent diabetes [6]. Non-insulin-dependent diabetes accounts for about 90% of cases (above 40-45 years). It occurs due to insulin resistance and β-cell dysfunction, leading to excessive gluconeogenesis (the synthesis of glucose from non-carbohydrate sources), glycolysis (the breakdown of glucose for energy), oxidative stress (a disparity within the production of harmful free radicals and the body's ability to fight against them), and the release of inflammatory mediators. Enzymes such as α-glucosidases, α-amylase [7], and dipeptidyl peptidase play a pivotal role in disrupting glucose homeostasis. These enzymes are responsible for reducing insulin secretion (the release of insulin from beta cells) and inactivating peroxisome proliferator-activated receptor gamma, a receptor involved in regulating glucose and lipid metabolism [8]. (iii) diabetes that originates during pregnancy and increases the risk of developing Type 2 Diabetes later in life is known as Gestational diabetes [9].

## 2 MANAGEMENT OF DIABETES MELLITUS

Effectively managing diabetes worldwide requires an intricate approach, integrating ongoing support, medical care, and patient education to prevent severe complications. Suddenly thirst, retinopathy, extreme hunger, frequent urination, and fatigue were observed in type-I and slowly in type II diabetic patients [10]. In treatment, to reduce the secretion of glucagon, an amylin analogue pramlintide and insulin were recommended. Simultaneously, physical activity, exercise, and a regular diet, along with glucose monitoring, are also needed to manage a hyperglycemic condition [11]. However, insulin therapy has limitations, including risks of weight gain, hypoglycemia, and localized injection reactions [12]. Various oral hypoglycemic agents are recommended for the treatment of diabetes, including metformin (a biguanide), Glimepiride, Glipizide, and Glibenclamide (sulfonylureas), Nateglinide and Repaglinide (meglitinides), Pioglitazone, and Rosiglitazone (thiazolidinediones), Vildagliptin, Saxagliptin, Sitagliptin, and Linagliptin (DPP-4 inhibitors), Dapagliflozin, Canagliflozin, and Empagliflozin (SGLT2 inhibitors), Acarbose (α-glucosidase inhibitor), Semaglutide (glucagon-like peptide-1 analogue), and Injectables such as insulin and exenatide, liraglutide (glucagon-like peptide-1 agonists) [13]. However, these medications are associated with various side effects. Biguanides surge in insulin sensitivity and suppress hepatic glucose production. These medications can cause central obesity, nausea, gastrointestinal discomfort, and a metallic taste [14]. Sulfonylureas and meglitinides activate insulin release from islets of Langerhans. Thiazolidinediones augment the sensitivity of insulin, stop insulin resistance, and lessen cardiovascular risks by reducing circulating fatty acids [15]. According to Chaudhury *et al.*, DPP-4 inhibitors stop the breakdown of incretin, a hormone that controls insulin secretion. Lifestyle modifications, including weight loss and physical activity, are foundational to T2D management but have limited patient adherence [16]. Dietary and lifestyle interventions are the first line of management for GDM, with insulin therapy as a secondary option when required [17]. Despite advancements in pharmacological treatments, long-term use of these agents is associated with side effects such as hypoglycemia, cardiovascular risks, and fluid retention, underscoring the need for alternative therapeutic strategies [18].

## 3 NATURAL PRODUCT IN MITIGATION OF DIABETES MELLITUS

Herbal medicines and their active compounds have gained recognition for their antidiabetic properties and reduced adverse effects. Plants are rich sources of secondary metabolites, which serve a variety of biological functions. Recently, there has been a growing interest in developing antidiabetic drugs from these natural compounds, particularly from plants traditionally used in alternative medicine practices for managing diabetes [19]. Secondary metabolites, including flavonoids, polyphenols, glycosides, tannins, terpenes, and alkaloids, offer diverse mechanisms to mitigate diabetes (Fig. **1**). These key ingredients can be applied directly, used in dry extract form, or formulated into different products to enhance patient compliance [20, 21]. As per ethnobotanical data, about 800 plants have been reported as antidiabetic drugs, and 150 secondary metabolites were isolated as having the potential of antidiabetic [22-27]. The herbal drugs contain a variety of compounds that can enhance pancreatic tissue activity, such as flavonoids [28], saponin glycosides [29], terpenes [30], phenols [31], and alkaloids [32, 33]. These compounds are effective through the following mechanisms of action.

- Enhancement of insulin secretion: flavonoids and alkaloids stimulate β-cell activity, increasing insulin release.
- Reduction of glucose absorption: compounds like saponins and tannins inhibit intestinal glucose uptake.
- Antioxidant activity: these metabolites neutralize free radicals, reducing oxidative stress-related β-cell damage.

The phytochemicals as a natural product are not beneficial in the prevention of diabetes mellitus but also important in the management of its complications. Among the above secondary metabolites, flavonoids are particularly promising due to their structural diversity and broad spectrum of biological activity, making them candidates for innovative therapeutic formulations. Many foods contain flavonoids, such as fruits, veggies, and their juices. A lot of citrus fruits, green veggies, and colored peppers like yellow and red peppers, are grown there. As a preventive approach, dietary therapy rich in flavonoids is highly recommended over allopathic medication due to its minimal side effects. Nutraceuticals, which are rich in flavonoids, offer a promising alternative for preventing and managing diabetes mellitus. These natural items are good for your health and don't have the adverse effects that are frequent with pharmaceutical drugs. However, it is important to note that while nutraceuticals are generally safe, they do have limitations, such as variability in flavonoid content, bioavailability issues, and the lack of established standardized dosages. Further research and public awareness are needed to enhance the understanding and adoption of flavonoid-rich dietary therapies for preventive health measures. This approach not only aligns with the principles of preventive medicine but also promotes overall health and well-being.

This study consolidates the applications of flavonoids in diabetes mellitus management, emphasizing their limits and examining several innovative formulations designed to improve their efficacy as antidiabetic drugs. The review highlights the effects and mechanisms of action of flavonoids, underlining their function in modulating blood glucose levels, enhancing insulin sensitivity, and mitigating oxidative stress. Flavonoids like rutin, quercetin, kaempferol, and naringenin offer a multifaceted strategy for managing diabetes by targeting various pathways implicated in its pathogenesis. Despite their various advantageous features, problems such as limited bioavailability, chemical instability, and vulnerability to degradation in adverse physiological conditions impede their clinical utilization. These constraints inhibit flavonoids from attaining optimal concentrations in target tissues, diminish their efficacy, and may lead to off-target consequences. Moreover, traditional distribution systems do not accommodate variations in glucose levels, heightening the risk of hypoglycemia and constraining therapeutic effectiveness.

Innovative drug delivery technologies, including nanoparticles (NPs), liposomes, and polymer-based carriers, are being developed to tackle these issues. These sophisticated methods seek to improve the stability and bioavailability of flavonoids, facilitating targeted delivery, enhancing absorption, and allowing regulated release. These systems provide a promising approach to maximize the antidiabetic potential of flavonoids by reducing potential side effects and enhancing therapeutic efficacy.

## 4 FLAVONOIDS AS A POTENTIAL SOURCE IN MITIGATION OF DIABETES MELLITUS

New developments enriched with flavonoids, such as phytomedicine, phytonutrients, nutraceuticals, and nutritional therapy, are readily available. Flavonoids, dietary polyphenols found naturally in various parts of plants, fruits, certain beverages, and vegetables, offer diverse applications, including antioxidative, anti-inflammatory, antidiabetic, anticancer, hypolipidemic, and neurodegenerative disease treatments [34, 35]. A study in the United States involving 200,000 individuals assessed the relationship between dietary intake of flavonoid subclasses and type II diabetes, demonstrating that increased consumption of anthocyanins from apples, blueberries, and pears reduces the risk of diabetes [36]. Chemically, flavonoids consist of 15 carbons, forming two aromatic rings (A and B) joined by three carbons conjugated with a heterocyclic oxygenated ring C (C6-C3-C6), categorized as diphenylpropanes. They can be further classified based on functional groups into flavanones, flavones, isoflavones, flavonols, anthocyanidins, and flavan-3-ols [37] (Fig. **2**). Flavonoids exert their antidiabetic effects through mechanisms such as peroxisome proliferator-activated receptor activation, insulin sensitivity enhancement, and modulation of gut glucose transport [38]. One possible explanation is that flavonoids' hydroxyl groups, α and β ketones, are responsible for most of their bioactivity [39]. In a study by Matsuda *et al.*, flavones and flavanols that contain 3-O and 4-O dihydroxy groups in ring B as well as those with a hydroxy group at the 7^th^ carbon, have strong activity against aldose reductase [40]. Furthermore, these interventions are more effective in reducing blood sugar levels when 2-3 double bonds are present; this leads to better glucose regulation and insulin sensitivity, which in turn provides a comprehensive approach to managing diabetes [41, 42]. Flavonoids have garnered significant attention in the field of pharmaceutical research and development. These are phytochemicals (flavonoids) that ameliorate diabetic conditions through various mechanisms of action (Fig. **3**).

Flavonoids have demonstrated significant potential in mitigating the development of diabetes and its associated complications. Their anti-diabetic properties are attributed to several key mechanisms [28, 43]. The following mechanism of actions through various common flavonoids are targeted and proven to be antidiabetic, as illustrated in Table **1** and Fig. (**4**). Enhancement of β-cell function: flavonoids improve insulin release by enhancing the functionality of β- cells in the pancreas [44]. Promotion of GLUT4 translocation: they facilitate the movement of Glucose transporter [45] T4, a protein essential for transporting glucose into cells [46]. Reduction of insulin resistance: flavonoids help decrease insulin resistance, contributing to better blood sugar management [47]. *Free radical scavenging:* They act as effective free radical scavengers, reducing oxidative stress that can exacerbate diabetes [48]. Inhibition of gluconeogenesis: Flavonoids inhibit gluconeogenesis in the liver by suppressing the appearance of key enzymes, for example, phosphoenolpyruvate carboxykinase and glucose-6-phosphatase. This reduces glucose production and availability in the liver [49]. Decreased apoptosis: They decrease cell death [apoptosis], which helps maintain the health of pancreatic cells involved in insulin production [50].

## 5 NANOTECHNOLOGY-BASED APPROACHES OF FLAVONOIDS TO COMBAT DIABETES

Since ancient times, humans have consumed various flavonoid-containing substances, either in their raw form or as conventional medicines. Flavonoids, as natural products, are large molecules with low solubility and exhibit unusual chemical variations, leading to diverse biological properties. Conventional dosage forms have several limitations, such as challenges with drug metabolism, reduced efficacy due to poor solubility, altered pharmacokinetic profiles, and a lack of target specificity. To address these formulation challenges, the application of nanotechnology has been proposed for flavonoids [73]. Nanotechnology consists of 10^-9^ to 10^-7^ sizes and is derived from Latin words. The nontechnology offers innovative solutions for diabetes management, particularly through the use of nano-sensors for quantum dot NPs to measure blood glucose levels and continuous glucose monitoring [74]. This research highlights the potential of nanotechnology in improving diabetes management practices. However, the poor solubility of flavonoids in water obstructs their therapeutic efficacy [75]. Therefore, different Nano formulations are recommended to improve the pharmacokinetic profiles of flavonoids. Flavonoids, when ingested orally, undergo a complex process starting from chewing, followed by the gastric phase where the acidic pH leads to hydrolysis of the food mass. As the food transitions from the stomach to the small intestine, the pH gradually rises from 6 to about 7.4 in the terminal ileum. Enzymes and biosurfactants released by the pancreas and bile further modify the composition of flavonoids. In the colon, gut microbiota utilizes the beneficial properties of these altered flavonoids and their derivatives. To prevent the degradation of phyto-markers at acidic pH or interaction with food matrices, novel formulations of flavonoids can be designed for target-specific mechanisms against various diseases, thereby increasing their absorption or bioavailability. Nanotechnological methods can address the pharmacokinetic challenges of polyphenols by developing lipid, inorganic, or polymer-based nano-formulations, whether natural or synthetic. These nano-formulations can encapsulate flavonoids in polymer or lipid barriers, retaining the active components in a complex structure to enhance their activity. (Fig. **5**) illustrates the development of the different types of nano-formulations of flavonoids for the modification and increase the efficacy of phytoconstituents or drug. The following are the different types of formulations such as:

Polymeric nanoparticles are defined as the particles varies in size from 1-100 nm and the development of NPs initiative was taken be National nanotechnology. This form of NPs is effective through the integration of surface ligand to the NPs, which leads to site-specific action and modifies the pharmacokinetic parameters of flavonoids [76, 77].

Liposomes are used to entrap the hydrophilic and lipophilic drugs in lipid layers containing cholesterol and phospholipids and release the drug in the targeted cell by merging the lipid layer with the cell membrane [78, 79].

Micelles are formulated using amphiphilic polymers to enhance the absorption of hydrophobic drugs and to protect functional groups by encapsulating them within the hydrophilic polymers [80].

Solid lipid nanoparticles are colloidal particles ranging in size from 50 to 1000 nm. They are formulated by incorporating phytocompounds coated with oppositely charged polymers, such as positively charged chitosan and negatively charged alginate. These formulations offer advantages over conventional dosage forms by improving bioavailability, increasing the release rate, and enhancing tolerability [81].

Nanogels or nanohydrogels are designed and developed by loading active compounds into PEG and methacrylic acid through the cross-linking of carboxyl and hydroxyl groups. These gels are effective because they can transform their structure by swelling or shrinking, depending on the pH and temperature of the surrounding media. Based on the pH, the gels either swell or release the phytoconstituents at the targeted site [82, 83].

The biosynthesis of metallic nanoparticles using medicinal plants has emerged as a promising and eco-friendly alternative to conventional chemical and physical synthesis methods, which are often associated with hazardous by-products. Plant extracts, particularly those rich in flavonoids and other active phytoconstituents, play a pivotal role in the synthesis of gold and silver nanoparticles through bio-reduction, stabilization, and bio-capping processes [24, 84-92]. These natural compounds not only enhance the stability of nanoparticles but also contribute to their bioactivity, making them highly suitable for therapeutic applications. The green synthesis of polymeric or metallic nanoparticles using herbal products has shown superior therapeutic efficacy compared to crude plant extracts [93]. This synergistic approach combining nanotechnology *i.e*, nanoparticles and plant-based medicine, highlights nanoparticles with flavonoid ligands, with their high encapsulation capacity, provide sustained release and prolonged hypoglycemic activity, offering a promising solution for diabetes control. The present condition of flavonoid-metal nanoparticle conjugates [94] and hybrids encompassing various synthesis methods and modifications of attributes like size, shape, surface charge, and stability [95]. The eco-friendly production of these nanoparticles not only aligns with sustainability goals but also represents a significant advancement in nanoscale medical research, particularly in the detection and treatment of illnesses. Numerous studies demonstrate the association of flavonoids with metal nanoparticles, primarily Ag, Au, and oxides of Fe, Zn, and Ti, yielding materials with notable physicochemical and biological features (Table **2**).

The Table **2** presents various nano-formulations developed using flavonoids to overcome diabetes mellitus and its complications. The activity of the developed nano-formulations has been assessed for reproducibility through *in vitro* and *in vivo* experiments. The Table **2** also illustrates the pathways through which these flavonoid formulations are effective as antidiabetic agents. These formulations aim to improve the efficacy of flavonoids by enhancing their solubility and absorption in the human body. This approach can increase clinical adherence and effectiveness by improving the bioavailability, absorption, and stability of flavonoids in managing diabetes and its associated vascular complications.

## 6 SYNERGISTIC AND ADDITIVE ANTIDIABETIC EFFECTS OF FLAVONOIDS AND THEIR POTENTIAL IN COMBINATION THERAPIES

Individual flavonoids have been widely reported to exhibit antidiabetic activity. For instance, Mahmoud *et al.* (2012) demonstrated that hesperidin and naringin attenuate hyperglycemia-mediated oxidative stress and pro-inflammatory cytokine production in diabetic rats [51]. Similarly, Koch *et al.* (2013) and Mukhopadhyay and Prajapati (2015) reported that dietary flavonoids, such as naringenin and quercetin, impair glucose metabolism, potentially by inhibiting hypothalamic insulin signaling, and highlighted the role of polymer-based carriers in improving quercetin bioavailability [120, 121]. Studies by Abdelmoaty *et al.* and Vaidya *et al.* confirmed the antidiabetic effects of quercetin and swertiamarin individually, showing their ability to regenerate β-cell function and promote insulin secretion [122, 123]. Al-Ishaq *et al.* (2019) further emphasized the cellular mechanisms of flavonoids in improving blood sugar levels, reinforcing their role as potent antidiabetic agents [28]. Moreover, Jaishree and Narsimha (2020) provided evidence of the synergistic antidiabetic effect of combining quercetin and swertiamarin, which enhanced β-cell survival and insulin release from the islets of Langerhans [124]. Recent work by Ali *et al.* (2024) demonstrated the additive effect of combining quercetin and kaempferol in diabetic rats. While these flavonoids target similar pathways, the combination improved the bioavailability of kaempferol, highlighting its potential benefits [125]. Imran *et al.* (2019) also underscored the significance of kaempferol’s bioavailability in enhancing therapeutic effects [126]. Additionally, Eldamarawi *et al.* (2020) reported a positive cumulative effect of combining quercetin with metformin, showing superior glucose-lowering activity, increased insulin levels, and reduced pro-inflammatory markers such as IL-1 and TNF-α, compared to individual treatments [127].

These studies illustrate the synergistic or additive antidiabetic effects of flavonoids when used in combination with other flavonoids or secondary metabolites. However, more scientific data is required to fully understand the synergistic mechanisms of flavonoids in diabetes management. While nano-formulations have proven effective in overcoming the limitations of flavonoids, such as poor bioavailability and stability, research on the synergistic effects of flavonoids in nano-formulations remains limited. Further investigations are necessary to explore the potential of nano-formulated flavonoid combinations to achieve enhanced antidiabetic activity.

## 7 PATENT INFORMATION OF FLAVONOIDS RICH NATURAL PRODUCTS IN MANAGEMENT OF DIABETES MELLITUS

The summary of patents on flavonoids highlights their role as antidiabetic agents through indirect pathways. Currently, there are no patents that specifically address the direct mechanisms by which flavonoids treat diabetes. However, many flavonoids have shown efficacy through indirect mechanisms, such as their antioxidant, anticancer, and stress-reducing activities, which can contribute to overall diabetes management. A summary on patents related to flavonoids that are effective in the management of diabetes mellitus is presented in Table **3**.

## 8 CHALLENGES AND LIMITATIONS

Flavonoids are predominantly found in the glycoside form, presenting a complex molecular structure. Their high molecular weight and size hinder absorption through the gastrointestinal tract, thereby impacting solubility, lipophilicity, and bioavailability [132]. These secondary plant metabolites are present in vegetables and fruits but in small quantities, making it challenging to incorporate them into a straightforward nutritional supplement. Given the diverse chemical structure and limited understanding of bioavailability, there is currently no established dosage for flavonoids. However, addressing challenges including solubility, cellular permeability, metabolic alterations, and excretion remains crucial [133]. Researchers are currently working on boosting the bioavailability of flavonoids by adding bioavailability enhancers, such as piperine, or specifically targeting absorption sites, enhancing metabolic stability, and optimizing glucose absorption from the intestine [134].

In pharmacokinetic studies, various challenges are observed in the absorption and metabolism of flavonoids. Flavonoids are primarily absorbed through the gastrointestinal system by exhibiting very less bioavailability, linked with the degradation of flavonoids during digestion. This degradation occurs due to the action of hydrolytic enzymes in the gastrointestinal tract, the inadequate transfer of flavonoids from the food matrix to the intestinal lumen, the composition of the microbiota, and the inefficient absorption by intestinal epithelial cells [135, 136]. The metabolism of flavonoids remains imperfectly documented due to their complex glycosidic structure. The hydrolysis or de-glycosylation primarily takes place in the small and large intestine through the action of enzymes such as β-glucosidases and Lactase-phlorizin hydrolase [137]. Enzymatic hydrolysis is the process by which glycone and aglycone components are separated from glycosides. The non-sugar component of flavonoids, referred to as free flavonoids, can enter the body by breaking down the pyrone ring structure. This breakdown is facilitated by specific microbes in the intestines, resulting in the production of phenylacetic and phenyl propionic acids, as well as other inactive substances [138]. Glycosidases catalyze the release of aglycones through various chemical reactions, including pyrone ring cleavage, hydroxylation, sulfonation, O-methylation, glucuronidation, and methylation facilitated by microbial modification in the intestines. Aglycones have a strong tendency to bind with blood proteins due to intermolecular linkages formed between the aglycone and albumin [139]. Consequently, complexes formed remain in the body for an extended period, leading to slow elimination. Therefore, an increased intake of flavonoids can result in their accumulation, increasing the likelihood of interaction with DNA and cellular proteins, thereby inducing toxicity such as genotoxicity and gene mutations.

## CONCLUSIONS AND FUTURE PROSPECTS

Fruits and vegetables are significant sources of flavonoids, rich in phytochemicals with immense therapeutic potential. With the rising global prevalence of diabetes mellitus, flavonoids are gaining attention for their role in managing this chronic disease through various biochemical pathways. The current study underscores the importance of developing new, efficient, safe, and low-toxicity medications for diabetes by utilizing flavonoids. The success of flavonoid-based formulations depends largely on patient compliance and their therapeutic effectiveness against targeted diseases.

Despite numerous advancements, including the formulation of antidiabetic nano-delivery systems, challenges persist in enhancing flavonoids' solubility, bioavailability, and absorption. Nanotechnology-based approaches, such as polymeric nanoparticles, liposomes, micelles, solid lipid nanoparticles, and nanogels, offer promising solutions to address these issues by improving pharmacokinetic profiles and enabling targeted delivery. This review emphasizes the need for extensive research to explore the individual potential of flavonoids and nano-formulations in combating diabetes and its complications. While significant progress has been made, a deeper understanding of their mechanisms of action is imperative to maximize clinical efficacy. Additionally, the therapeutic window of flavonoids must be carefully optimized to balance efficacy and safety.

Future studies should focus on overcoming pharmacokinetic challenges, refining nanotechnology-driven delivery systems, and investigating the molecular pathways involved in flavonoid activity. This will enable the development of innovative, patient-centric, and sustainable solutions for diabetes management, paving the way for improved clinical outcomes and reduced disease burden worldwide.

## Figures and Tables

**Fig. (1):**
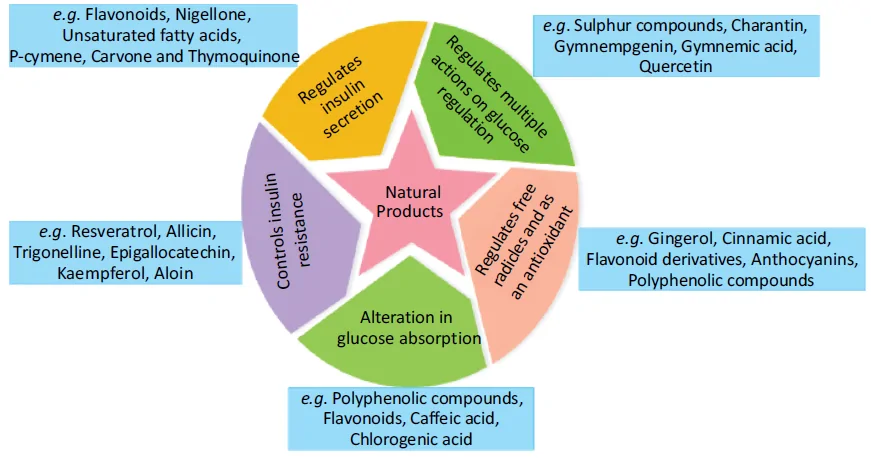
Natural product as potential source in mitigation of diabetes mellitus.

**Fig. (2):**
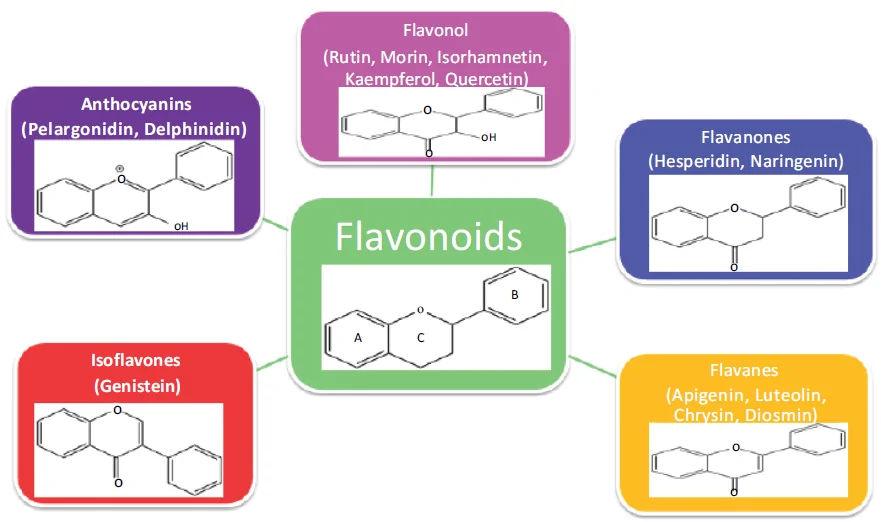
Classification of flavonoids.

**Fig. (3):**
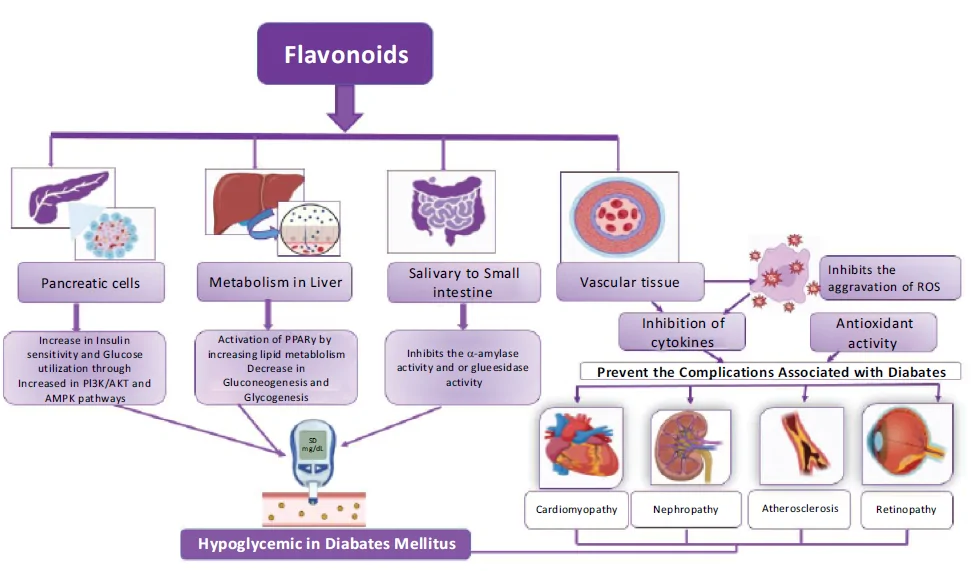
Amelioration of antidiabetic activity of flavonoids through various mechanism of action.

**Fig. (4):**
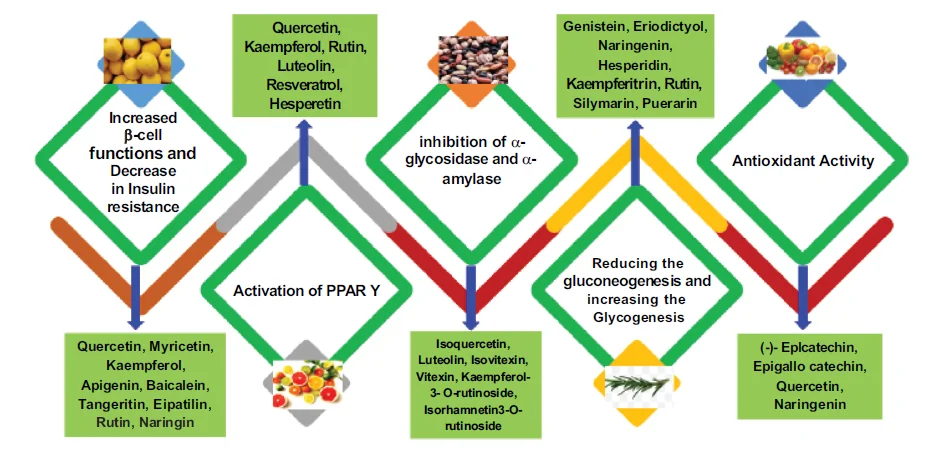
Examples of common flavonoids for the targeted mechanism of action.

**Fig. (5):**
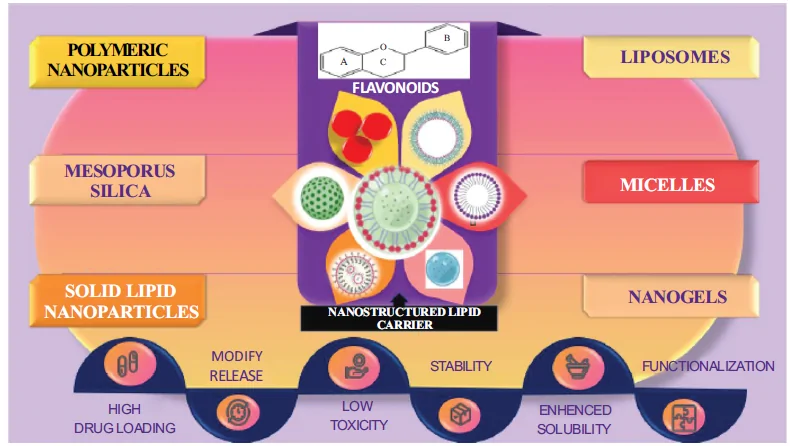
Different types of nano formulation by incorporating flavonoids.

**Table 1:** Different flavonoids sources along with their mechanism of actions as an antidiabetic.

**Phytochemicals**	**Sources**	**Category of Flavonoids**	**Mechanism for Antidiabetic**	**References**
Hesperidin and Naringin	Naringin obtained from in citrus fruits, such as tomatoes, oranges, grapefruits and berries	Flavanones, also referred to as dihydroflavones, are distinguished by an oxidized, saturated carbon ring. It is known as 5,7,4’-Trihydroxyflavanone, a saturated oxidized aglycone.	Enhance the capacities of antioxidant enzymes, elevate levels of NO and MDA in the serum and liver, and boost the activity of the antioxidant defence system.	[51]
	Hesperidine mainly obtained from citrus fruits, such as oranges, lemons, limes, tomatoes and cherries	Found as Flavanones category and found as saturated oxidized aglycone in the form of 5,7,3’-Trihydroxy-4’-methoxyflavanone.		
Hesperidin, quercetin	Quercetin obtained from mostly in various fruits, apples, berries, seeds of tomatoes, fennel, nuts, onions, tea leaves, broccoli, pepper, lovage, shallots and flowers.	Flavonols are represented by an unsaturated carbon ring between carbons 2 and 3, which is oxidized at carbon 4 and hydroxylated at carbon 3.	Autooxidation of glucose by aggregating the antioxidant enzymes and falling the lipid peroxidation in the liver.	[52]
**Increased β-cell Functions and Decrease in Insulin Resistance**				
Puerarin	Extracted from the Kudzu root and *Pueraria* genus	Isoflavones	In order to protect the mitochondria in muscle from oxidative stress it is necessary to decrease the phosphorylation of p66Shc and boost the expression levels of superoxide dismutase-2 [SOD], SIRT3, UCP2, and UCP3. This will enhance the efficiency of antioxidant defence and reduce the generation of free radicals.	[53]
Anthocyanins	Anthocyanins are water-soluble pigments responsible for the red, purple, or blue coloration of fruits, present in foods (jams, jelly candies, fresh sausages), beverages (yogurts, juices), vegetables, flowers, and certain grains. It also found in red and purple berries, grapes, apples, plums, and cabbage.	Polyphenolic flavonoids having C6-C3-C6-skeleton	Protects hepatic cells from oxidative deterioration by increasing superoxide dismutase antioxidant activity, reducing reactive oxygen species generation, and boosting cell viability. Blocks NF-κB and MAPK activation.	[54]
Myricetin	It found nuts, vegetables, herbs, fruits, Garlic, lotus root, berries, plants together with beverages, such as fruit (Citrus), tea, wine. It also found in *Rosa canina* L. (rosa hip), *Portulaca oleracea* L. and *Urtica dioica* L. (nettle) medicinal plants	It is basically flavanols derivatives and Myricetin glycosides comprise myricetin-3-O-[4″-acetyl]-α-l-arabinopyranoside, myricetin-3-O-[3″-acetyl]-α-l-arabinopyranoside, myricetin-3-O-β-d-galactopyranoside, myricetin-3-O-α-l-rhamnopyranoside, myricetin-3-O-β-d-xylopyranoside, myricetin-3-O-α-l-arabinofuranoside.	Prevented lipoxygenase activity.	[55]
Naringenin	Same as Serial no 1A	Same as serial no 1A	Decreases inflammation-mediated nitric oxide overproduction and boosts endogenous antioxidant status during hyperglycaemia so it is proven to be anti-inflammatory and antioxidant.	[56]
Puerarin	Same as Serial no 3	Same as serial no 3	Enhances insulin signalling and regulates the B-AMPK/ACC pathway by stimulating the μ-opioid receptor in skeletal muscle.	[57, 58]
Baicalin	Isolated from Bael fruit, the roots of *Scutellaria baicalensis* and fruits of *Oroxylum indicum* L.	As a Flavones and available as 5,6,7-Dihydroxyflavone aglycone	Activating the GALR2-GLUT4 signal pathway prevents insulin resistance and metabolic abnormalities.	[59]
Anthocyanins	Same as Serial no 4	Same as serial no 4	Triggering the IRS-1/AKT pathways.	[60]
Epigallocatechin gallate [EGC], quercetin	It is obtained from the teas, pome fruits, like pears, apples, cocoa products and juices of prune	EGC is a flavanol group of polyphenols	Addressing redox imbalance and mitochondrial malfunction to avoid TNF-α-induced insulin signalling pathway blockage	[61]
Naringenin	Same as Serial no 1A	Same as serial no 1A	Endoplasmic reticulum stress, IRE1 downstream gene expression, and IRE1 expression were lowered by HCVCP, improving insulin resistance.	[62, 63]
Scutellarein	It is extracted from *Scutellaria* plants, *Erigeron* plants, *Centaurea* plants, *Opuntia* plants, and *Anaphalis* plants	Flavone derivative	Amplification of insulin signalling pathways and AMP-activated protein kinase.	[64]
Luteolin	Obtained from celery, green pepper, parsley, broccoli, apple skins, onion leaves, carrots, cabbages, chamomile tea and *Chrysanthemum* flowers	Available as flavone derivative and available in the form of 3′,4′,5,7-tetrahydroxyflavone	Activates the PI3K/AKT signalling pathway.	[65]
Biochanin A	It is obtained from red clover, cabbage, alfalfa and *Trifolium pratense*	Isoflavone available as 5,7-dihydroxy-4’-methoxy-isoflavone	Increasing the phosphorylation levels of the skeletal muscle proteins GLUT4, PI3K, IRS-1, and p-AKT.	[66]
**Regulating Glucose and Lipid Metabolism**				
Rutin	Rutin is obtained and extracted from plants, such as grapes, oranges, lemons, buckwheat peaches, and limes	Rutins, also known as glycosylated quercetin, quercetin-3-O-rutinoside and sophorin.	Reduction in various lipid parameters such as total cholesterol, triglycerides and low density lipoproteins.	[67]
Silymarin	Obtained from *Silybum marianum*	It is a type of flavonolignan available as an isomeric mixture of the flavonolignans silydianin and silychristin, which occur in two diastereoisomeric forms, A and B, as well as silybin and isosilybin, both of which also have two diastereoisomers: silybin A and B, and isosilybin A and B.	Improve insulin resistance in the liver, increase glycogen content, and decrease lipid build-up.	[68, 69]
**Regulate the Carbohydrates Metabolism by Reduction in the Gluconeogenesis and Increasing the Glycogenesis**				
Kaempferol	Found in green Tea, broccoli, beans, strawberries, apples, yellow fruits (citrus), and grapes. And also obtained from medicinal herbs such as *Aloe vera*, *Embelia ribes*, Delile, *Ginkgo biloba *L, *Euphorbia pekinensis* Rupr, and *Rosmarinus officinalis*	Present in the form of [3,4′,5,7-tetrahydroxyflavone] and type of flavonol derivative	Decreased glucose uptake in the liver and impaired glucose utilisation in skeletal muscle.	[49]
Puerarin	Same as serial no 3	Same as serial no 3	Enhance the phosphorylation of fork-head box protein O1 by activating the PI3K/Akt signalling pathway, which in turn reduces the production of phosphoenolpyruvate carboxykinase and glucose-6-phosphatase, so reducing gluconeogenesis.	[70]
**Reduces the Diabetic Complications such as Wound Healing, Diabetes Retinopathy, Diabetes Nephropathy, Diabetes Cardiomyopathy**				
Silymarin, kaempferol, quercetin, puerarin, Luteolin, and rutin	Same as serial no 16, 17, 2, 3, 13 and 15	Same as serial no 16, 17, 2, 3, 13 and 15	By inhibiting NF-κB pathways, reducing the synthesis of advanced oxidation protein products, and significantly decreasing collagen deposition and cardiac fibrosis in diabetic rats, blood glucose levels were lowered.	[71, 72]

**Table 2:** Nano formulations of flavonoids for the management of diabetes mellitus.

**Phyto-markers**	**Formulations**	**Mode of Action**	**References**
Luteolin	Luteolin/ZnO NPs	By elevating and improving the insulin levels, Antioxidant activity. Antioxidant activity is enhanced by increasing insulin levels. For the purpose of preventing diabetic neuropathy, the formulation reduces inflammatory damage *via* regulating redox-sensitive signal transduction pathways.	[96]
	Luteolin/zinc oxide nanoparticles [Lut/ZnO NPs]	Improving insulin sensitivity, enhancing antioxidant levels, and modulating the insulin signaling system. The Luteolin/ZnO nano-dispersions enhanced the expression of proteins associated with the insulin signaling pathway in the liver, including IRS, PI3K, and AKT.	[97]
Rutin	Chitosan-alginate NPs loaded by incorporating rutin	Increase the bioavailability of Rutin and significant increase in glucose uptake and exhibited antidiabetic activity.	[98]
Myricetin	Myricetin-loaded solid lipid NPs	Revealed a remarkable therapeutic impact in reversing hyperglycemia, impaired glucose uptake by myotubes, insulin resistance, and pancreatic apoptosis in different animal models.	[99]
Myricetin	Myricetin encapsulated within chitosan NPs	Showed better glycemic control.	[100]
Quercetin	Quercetin-NPs prepared by using Eudragit L-100	Antidiabetic effect.	[101]
	Superparamagnetic iron oxide NPs linked with quercetin	Enhanced cognitive impairment caused by diabetes in rats.	[102]
	Poly lactic acid/polyethylene glycol with quercetin nanocarriers	Used in diabetes complications such as diabetic nephropathy.	[103]
	Quercetin-loaded advanced nano-formulations	Antidiabetic effect.	[104]
Apigenin	Apigenin-loaded nanoliposomes	Protect against apoptosis in rats with diabetes cardiomyopathy.	[105]
Naringenin	Bovine β-casein micelles	Crosslinking of naringenin with beta casein micelles showed stable delivery systems.	[106]
Pelargonidin	PLGA NPs formulated by incorporating pelargonidin	Formulation reduces hyperglycemia-induced apoptosis, DNA damage, oxidative stress, and glucose utilization in L6 skeletal muscle cells more effectively and at a lower dose (~10-fold less) than natural pelargonidin.	[107, 108]
Baicalin	Baicalin-loaded nanoemulsion	Used in treatment of diabetes type II.	[109]
Combination of bitter gourd, guava, amla, fenugreek, jamun, gymnema	ADJ6	The high concentration of phytochemical ingredients, such as total phenols, flavonoids, and vitamin C, within the extract effectively inhibits the enzymes α-amylase and α-glucosidase.	[110]
Catechin	Catechin-grafted chitosan NPs	Effective as an antioxidant in antidiabetic patients.	[111]
Resveratrol	Multilayered nanoliposomes prepared by PEG-amalgamated	Effective as antioxidant and improving the diabetic condition by changing the glycemic status.	[112]
EGCG	Epigallocatechin gallate-loaded cationic lipid NPs	Showed improvement in the conditions of diabetes.	[113]
Gallic acid	Combination of insulin and gallic-acid NPs	The PI3K/Akt/GLUT4 pathway is activated *in vitro* to improve the liver's utilization of glucose.	[114]
*Olea europaea* extract	Chitosan coated liposomes	α-glucosidase and α-amylase inhibition assays and antioxidant activity.	[115]
*Argyreia pierreana* and *Matelea denticulata*	Micellar nano-formulations	Antioxidant and antihyperlipidemic activity.	[116]
*Trachyspermum ammi*	Nanosuspensions	Inhibition of α-amylase, and α-glucosidase.	[117]
Isoorientin	Isoorientin loaded silver nanoparticles (AgNPs-Iso)	AgNPs-Iso also inhibited alpha-glucosidase and pancreatic lipase, which are associated with the onset of type II diabetes and obesity, respectively.	[118]
*Clitoria ternatea* extract	Magnetic (cobalt oxide) and noble metal (gold) nanoparticles (Au NPs, and Co_3_O_4_ NPs) conjugated with flavonoid rich extract of *Clitoria*	Significant reduction in the diabetic enzyme levels (α-glucosidase, α-amylase, and xanthine oxidase) and enhancing the antioxidant activity.	[119]

**Table 3:** Patents related to flavonoids that are effective in management of diabetes mellitus.

**Patent ID**	**Title**	**Invention**	**Status**	**References**
WO2024110926A1	Nutraceutical supplement based on polyphenol matrix	A polyphenolic matrix, suitable as a nutraceutical supplement consisting of ellagic acid, hesperidine, brutieridin, naringin, neohesperidin, brutieridin, melitidin, neoeriocitrin, and melitidin, proved in diabetes treatment by lowering diabetes-induced obesity and other lifestyle disorders.	Active	[128]
US9381223B2	Methods for inhibiting advanced glycation end product production, inhibiting fibroblast apoptosis, and/or promoting human fibroblast-collagen grating formulation using cherry blossom and cherry leaf extract	Polyphenols are the major phytochemicals present in cherry trees. Along with polyphenols, trees also contain phenylpropanoids and flavonoid glycosides, which are responsible for inhibiting AGE production and promoting the formation of fibroblast-collagen matrix.	Active	[129]
CN112450435A	Health food for reducing blood sugar and blood fat and preparation method thereof	The invention is related to health food for monitoring the blood sugar with exhibiting antioxidant activity. The health food comprised of different poly-phenolic plant, alpha-amylase inhibitor, aloe extract, fruit extract, cassia seed extract and konjac flour showed antidiabetic activity by reducing blood glucose, inhibiting α amylase activity and hydrolysis of polysaccharides.	Withdrawn	[130]
KR102072612B1	Compositions and methods for improving mitochondrial function and treating neurodegenerative diseases and cognitive disorders	In this invention role of ellagitannins as a polyphenolic have potential role in antioxidant, anti-stress, anti-diabetic and anticancer by *in vivo* and *in vitro* activity.	Active	[131]

## LIST OF ABBREVIATIONS

AgNPs-Iso: Isoorientin Loaded Cilver Nanoparticles
NPs: Nanoparticles
SOD: Superoxide Dismutase-2
